# The Strange Case of Dr. Bruno

**Published:** 1906-12

**Authors:** 


THE STRANGE CASE OF DR. BRUNO. The publishers write me: "Never before has a book received so much notice and in so short a time as has yours. In twenty-four hours after it was in the hands of the *Times Book Review*, the following very complimentary notice appeared":

A DOCTOR'S STORY

(From *New York Times*, November 17, 1906.)

"The Strange Case of Dr. Bruno" (New York: Guarantee Publishing Company). The case is ingeniously invented. A gentleman of wealth and German descent and education, who has his happiness wrecked by a conspiracy of cruel fate, goes to school to the mud wasp or "dirt-dauber" to find a drug which will suspend all the faculties in such a fashion as to preserve life under the appearance of death. Thus the mud wasp preserves the spider as fresh meat for her young, and Dr. Bruno obtains a fluid chemically similar to that in the sac behind the wasp's sting, which he tries upon mice, pigs and monkeys, till he is sure enough of his results to experiment upon himself.

He times the dose for six months and one hour precisely and lies down to pass that space in a state of animation so suspended that, as there shall be little or no waste, so there shall be no need of sustenance. The outcome of the experiment appears in the story, which deals very tragically with the passion of love and involves two beautiful women and two very handsome men. The scene is laid in New Orleans and in the city of Jackson, Miss., and the author, F. E. Daniel, M. D., has availed himself of all the tricks of melodrama to spice the dish of his medical fancy. The medical fancies are better than the rest—have, in fact, a quite convincing air of verisimilitude.

The New York *Commercial* (November 20) says:

A few years ago the *New Orleans Picayune* published a remarkable story of a man who was lying in a condition closely resembling death in the house and under the care of an eminent oculist and an equally eminent physician, both of New Orleans. Some months after the paper again referred to the case, the subject then having been in a comatose state for nearly six months. By this time the facts had become known to the leading physicians of the city, who were much puzzled and were watching for the outcome with great interest. The case was discussed then and is still referred to as "The Strange Case of Dr. Bruno."

The oculist has since died, leaving among his manuscripts a full account of the affair, and his heir and executor, Dr. Ferdinand E. Daniel, has given it to the world. *A stranger story was never penned.*

Dr. Bruno, who, after years of study, tried the experiment upon himself of suspending animation without destroying life, was a fellow student of the oculist at Heidelberg, and they became intimate friends. He was quite successful in the experiment, and lay in a comatose state without food or water for six months. He has left his story in manuscript. Aside from the experiment, Dr. Bruno's life was curious and eventful, containing a romance which the reader will eagerly follow. "The Strange Case of Dr. Bruno" is published by the Guarantee Publishing Company. Dr. Daniel was president of the American International Congress on Tuberculosis, which closed its sessions at the Hotel Astor last week.

The following appeared in *The Bookseller, News Dealer and Stationer:*

"The Strange Case of Dr. Bruno": A romance of love smelted in the crucible of science. No more wonderful and more thrilling tale has ever been woven in the history of literature than this story of Dr. Baron von Bruno, Jr., who discovered the secret of longevity through a compound which produces sleep for any number of months, and the harmless suspension of all physical functions.

This book takes an enduring place in literature and science.

Of intense interest to all classes.

An interview with the author of "The Strange Case of Dr. Bruno" by Mr. Selig, of the *New York Evening Journal:*

SCHEME TO HAVE CONDEMNED CRIMINALS TURNED OVER TO SCIENCE.

The use of the condemned murderer for scientific purposes, as Pasteur used pigs, rabbits and horses, is the long cherished ambition of Dr. Ferdinand Eugene Daniel, of Austin, Texas, one of the foremost physicians of the country.

Seated in the lobby of the Hotel Gerard, the doctor went into details of his startling ideas. A man of more than six feet in height, faultlessly dressed, he looked the typical savant. He said:

"The condemned criminal is a waste product, the scum that rises to the surface in the ebullition of the social cauldron, and, like all other waste products, should be utilized by science in the interest of the human race.

"I maintain and I know that there are certain infectious diseases that experimenting with on a rabbit or a pig or dog could be of no earthly use. We must have a human being to experiment on.

"A condemned murder is of no use to society. Why not turn him over to a regularly appointed State's physician to have him inoculated for the benefit and the enlightenment of the human race. Inject into him various disease germs, watch their progress, and when through with him, inject about ten drops of prussic acid into the veins of his arms and he will die a painless death.

"The condemned murderer should not be handed over to the medical men until he has been taken upon the scaffold or in the electric chair. When all hope is gone, then turn him over to them and let him be of some use to humanity."

Dr. Daniel has embodied his views in a book called "The strange Case of Dr. Bruno," one of the literary sensations of the year. Every incident in this remarkable book has been taken from cases that have happened in real life, and the hero of the book, Dr. Bruno, simply represents the views of the author.

"The theories of Dr. Bruno, as expressed in the book," said Dr. Daniel, "are my own only up to the point where the theories as expressed by this German physician are acted on, and the rest then becomes fiction. The origin of the book was a 'dirt-dauber,' or a solitary wasp.

"Now, if a plain, ordinary wasp can be in possession of a poison whereby she can put in a dormant state a creature heavier than herself, why can not man come into possession of a drug whereby man could be put in a dormant state and still be preserved without any bad effects to himself? Why can't I do to a man what the wasp did to the spider?

"When the people will read of my theories they will say that I am an inhuman monster. Do you know that I have been president of the Humane Society in my city, and that I am interested in all that is humane and that will ameliorate the conditions of the public? But I say again, and say it very emphatically, that the time will come when an enlightened people will wake up to the fact that their medical needs must be recognized by the highest officials of the land, and that a law will be eventually passed giving the care of condemned murderers over to physicians for scientific research."

"THE STRANGE CASE OF DR. BRUNO" is being dramatized.

